# Involvement of K_V_3.4 Channel in Parkinson’s Disease: A Key Player in the Control of Midbrain and Striatum Differential Vulnerability during Disease Progression?

**DOI:** 10.3390/antiox13080999

**Published:** 2024-08-18

**Authors:** Giorgia Magliocca, Emilia Esposito, Michele Tufano, Ilaria Piccialli, Valentina Rubino, Valentina Tedeschi, Maria Jose Sisalli, Flavia Carriero, Giuseppina Ruggiero, Agnese Secondo, Lucio Annunziato, Antonella Scorziello, Anna Pannaccione

**Affiliations:** 1 Division of Pharmacology, Department of Neuroscience, Reproductive and Dentistry Sciences, School of Medicine, Federico II University of Naples, Via Pansini, 5, 80131 Naples, Italy; giorgia.magliocca@unina.it (G.M.); emilia.esposito13@studenti.unina.it (E.E.); michele.tufano2@unina.it (M.T.); ilaria.piccialli@unina.it (I.P.); valentina.tedeschi@unina.it (V.T.); secondo@unina.it (A.S.); 2 Department of Translational Medical Sciences, University of Naples Federico II, 80131 Naples, Italy; valentina.rubino@unina.it (V.R.); mariajose.sisalli@unina.it (M.J.S.); giruggie@unina.it (G.R.); 3 Department of Sciences, University of Basilicata, 85100 Potenza, Italy; flavia.carriero@unibas.it; 4 SYNLAB SDN, Via Emanuele Gianturco, 113, 80143 Naples, Italy; lannunzi@unina.it

**Keywords:** Parkinson’s disease, K_V_3.4 channel, A53T mice, ROS, calcium dyshomeostasis, astrocyte

## Abstract

Parkinson’s disease (PD), the second most common neurodegenerative disease in the elderly, is characterized by selective loss of dopaminergic neurons and accumulation of α-synuclein (α-syn), mitochondrial dysfunction, Ca^2+^ dyshomeostasis, and neuroinflammation. Since current treatments for PD merely address symptoms, there is an urgent need to identify the PD pathophysiological mechanisms to develop better therapies. Increasing evidence has identified K_V_3.4, a ROS-sensitive K_V_ channel carrying fast-inactivating currents, as a potential therapeutic target against neurodegeneration. In fact, it has been hypothesized that K_V_3.4 channels could play a role in PD etiopathogenesis, controlling astrocytic activation and detrimental pathways in A53T mice, a well-known model of familial PD. Here, we showed that the A53T midbrain, primarily involved in the initial phase of PD pathogenesis, displayed an early upregulation of the K_V_3.4 channel at 4 months, followed by its reduction at 12 months, compared with age-matched WT. On the other hand, in the A53T striatum, the expression of K_V_3.4 remained high at 12 months, decreasing thereafter, in 16-month-old mice. The proteomic profile highlighted a different detrimental phenotype in A53T brain areas. In fact, the A53T striatum and midbrain differently expressed neuroprotective/detrimental pathways, with the variation of astrocytic p27^kip1^, XIAP, and Smac/DIABLO expression. Of note, a switch from protective to detrimental phenotype was characterized by the upregulation of Smac/DIABLO and downregulation of p27^kip1^ and XIAP. This occurred earlier in the A53T midbrain, at 12 months, compared with the striatum proteomic profile. In accordance, an upregulation of Smac/DIABLO and a downregulation of p27^kip1^ occurred in the A53T striatum only at 16 months, showing the slowest involvement of this brain area. Of interest, HIF-1α overexpression was associated with the detrimental profile in midbrain and its major vulnerability. At the cellular level, patch-clamp recordings revealed that primary A53T striatum astrocytes showed hyperpolarized resting membrane potentials and lower firing frequency associated with K_V_3.4 ROS-dependent hyperactivity, whereas primary A53T midbrain astrocytes displayed a depolarized resting membrane potential accompanied by a slight increase of K_V_3.4 currents. Accordingly, intracellular Ca^2+^ homeostasis was significantly altered in A53T midbrain astrocytes, in which the ER Ca^2+^ level was lower than in A53T striatum astrocytes and the respective littermate controls. Collectively, these results suggest that the early K_V_3.4 overexpression and ROS-dependent hyperactivation in astrocytes could take part in the different vulnerabilities of midbrain and striatum, highlighting astrocytic K_V_3.4 as a possible new therapeutic target in PD.

## 1. Introduction

Parkinson’s disease (PD), a chronic and progressive neurodegenerative disorder, is characterized by bradykinesia, rigidity, difficulty balancing, and non-motor manifestations [1,2]. The two major PD neuropathological hallmarks are the premature selective loss of midbrain dopaminergic neurons in the *substantia nigra pars compacta* and the accumulation of neurotoxic Lewy bodies, which mainly consist of misfolded α-synuclein (α-syn) [3,4]. The exact etiology underlying the selective dopaminergic neurodegeneration is not yet completely understood, but it is assumed to be the result of a complex interplay of genetic and environmental risk factors [5]. Since current treatments for PD merely address symptoms, there is an urgent need to identify the PD pathophysiological basis for developing better therapies [6].

Recently, a growing number of studies propose potassium (K^+^) dysfunction, mainly caused by an impairment of plasma membrane voltage-gated potassium (K_V_) channels, as a key player in the pathogenesis and progression of PD due to its role in regulating neuronal excitability, neurodegeneration, and neuroinflammation [7,8,9,10].

K_V_ channels, the most widely distributed transmembrane channels, play in neuronal cells important roles in regulating numerous neurophysiological functions, including resting membrane potential, spontaneous firing rate, and neurodegenerative and neuroinflammatory processes [11,12]. Their involvement in neurodegenerative pathways is supported by the evidence that the inhibition of K^+^ efflux, induced by a wide range of K^+^ channel blockers or the increase in extracellular K^+^ concentration, fully prevents cell death [13,14,15].

The inhibition of K_V_ channels enhances the spontaneous firing frequency of nigral dopaminergic neurons, induces a transition from tonic firing to burst discharge, and promotes the release of dopamine (DA) in the striatum. In particular, the inhibition of fast-inactivating K^+^ channels, by a well-known blocker 4-aminopyridine (4-AP), results in depolarization and an increase in excitability converting the neuronal tonic firing mode to burst firing, thus enhancing the release of DA [16,17]. Moreover, 4-AP increases excitability and activates silence cells (12,18), prolongs active periods, and augments dendritic Ca^2+^ spikes [18]. Of note, 4-AP has been approved by the Food and Drug Administration, FDA, as a drug for the treatment of patients with multiple sclerosis [19]. Recent findings have reported changes of neuronal K_V_4.3 channel expression in PD animal models [7]. In particular, Subramaniam et al. [7] identified an increased action potential firing rates of DA neurons of the ventral midbrain in A53T mice at the late phase (at 8 months). In addition, the frequency increase is caused by a redox-mediated impairment of fast-inactivating neuronal Kv4.3 channels.

Likewise, the inhibition of delayed channels prevents neuronal apoptosis and improves motor coordination [20]. In 1-methyl-2-phenyl-1,2,3,6-tetrahydropyridine (MPTP)-treated PD mouse models, an overexpression of K_V_2.1 channels associated with the DA neuron loss in the *substantia nigra* and striatum DA terminals has been observed [20]. Consistently, the inhibition of the K_V_2.1 channel by a specific blocker, guangxitoxin-1E, protects nigrostriatal projections against MPTP/MPP^+^ insult and facilitates the recovery of motor coordination. Moreover, it has been observed that 4-AP and tetraethylammonium (TEA), a potent blocker of delayed rectifier K^+^ channels [11], have significant effects in the treatment of 6-hydroxydopamine (6-OHDA)-induced PD depending on the dose and degree of severity, suggesting their potential use in the treatment of PD [12]. By contrast, in astrocytes, the blockade of K_V_ channels reduces their ability to repolarize and the influx of Ca^2+^ [21].

Astrocytes are considered not electrically silent cells, displaying hyperpolarized resting membrane potentials that are critical for enabling and regulating neurotransmitter reuptake and K^+^ buffering [22,23,24]. However, the functional implications of K_V_ channels in astrocyte function have not been fully elucidated.

Recently, the K_V_3.4 channel, a reactive oxygen species (ROS)-sensitive channel carrying a fast-inactivating current and blockade by 4-AP and TEA [25,26], is emerging as a new target candidate in Alzheimer’s disease (AD) [27,28,29,30,31]. Of note, an overexpression of K_V_3.4 channels has been observed in the early and advanced stages of AD, suggesting that these channels might take part in the neurodegenerative processes occurring in AD human brains [27]. In line with this, an overexpression of the K_V_3.4 channel, induced by amyloid-β (Aβ), has been observed in neurons and astrocytes in an AD model during the early and late stages [28,29,30]. The pharmacological inhibition or genetic knockout of the Aβ_1-42_-induced hyperfunctional K_V_3.4 channel displays a distinct role in neurons and astrocytes [28,29,30,32]. Indeed, in neurons, the blockage of K_V_3.4 channels prevents neuronal cell death induced by neurotoxic Aβ, whereas in astrocytes it restores spontaneous [Ca^2+^]_i_ transients [28,29,30,32].

Despite major neuropathological differences, common pathways have been recognized for AD and PD, including Aβ and α-syn misfolding, mitochondrial dysfunctions, oxidative stress, Ca^2+^ dyshomeostasis, impairment of the lysosomal-autophagy pathway, and neuroinflammation. Therefore, it has been hypothesized that K_V_3.4 channels could play a role in PD etiopathogenesis controlling astrocytic activation and detrimental pathways in A53T mice, a well-known model of familial PD. To this aim, the putative role of K_V_3.4 channels as a causative protein involved in the different vulnerabilities of the midbrain and striatum in PD progression is investigated in A53T mice.

## 2. Materials and Methods

### 2.1. Animals

Transgenic mice expressing human A53T-α-synuclein (A53T) under the control of a prion promoter (PrP-SNCA*A53t) [33] were purchased from the Jackson Laboratory (Bar Harbor, ME, USA). Hemizygous A53T mice were bred on a mixed C57Bl/6 × C3H background to obtain transgenic littermates at 6, 12, and 16 months (#5 mice for each group), as well as non-transgenic littermates #7 mice at 6 months and #5 mice for groups at 12 and 16 months [34]. Transgenic mice were identified by RT-PCR [2]. Animals were housed as previous described [2]. Experiments were performed in accordance with the International Guidelines for Animal Research and approved by the Animal Care and Use Committee of “Federico II” University of Naples, Italy (OPBS Centro Servizi Veterinari) on 21 August 2023, No. 738/23.

### 2.2. Primary Astrocytes from A53T-α-Syn Mice

Primary midbrain and striatal astrocyte cultures were obtained from 1/2-day-old A53T-α-synuclein and WT newborn pups (P1–2), as previously described [2].

### 2.3. Western Blot Analysis

Midbrain, striatum and primary astrocytes were lysed in RIPA buffer according to the Proteintech protocol (Tris-HCL 50mM, pH 7.4; NaCl 150mM; Triton X-100 1%; Sodium deoxylcholate 0.5%; SDS 0.1%; EDTA 1mM; NaF 10mM; PMSF 1mM; Na_3_VO_4_ 1 mM; and protease inhibitor cocktail Roche). Homogenates were centrifuged at 10,000 rpm for 20 min at 4 °C, and the supernatant was used to perform Western blot analysis as previously described [2]. The membranes were blocked in a bovine serum albumin-based buffer (3% BSA in TBS containing 0.1% Tween^®^20) for 1 h at room temperature (RT). The membranes were incubated overnight at 4 °C in the blocking buffer with 1:1000 anti-K_V_3.4 (rabbit polyclonal; Alomone Labs, Jerusalem, Israel; APC-019), 1:1000 anti-Smac/Diablo (mouse monoclonal; Abcam, Cambridge, UK; ab111893), 1:1000 anti-p27^kip1^ (mouse monoclonal; Antibodies.com, Cambridge, UK; A251279), 1:5000 anti-XIAP (mouse monoclonal; Proteintech Biotechnology, Rosemont, IL, USA; 66800-1-Ig), 1:1000 anti-HIF-1α (mouse monoclonal; Santa Cruz Biotechnology, Inc., Dallas, TX, USA; Sc13515), 1:10,000 anti-β-actin-peroxidase (mouse monoclonal; Sigma, Milan, Italy; A3854), and 1:1000 anti-tyrosine hydroxylase (TH; mouse monoclonal; Sigma, Milan, Italy; T1299) antibodies [2]. Immunoreactivity was detected by chemiluminescence with a Chemidoc machine (Bio-Rad, Hercules, California, USA), and band quantification was performed using ImageJ software (NIH, Bethesda, MD, USA).

### 2.4. Apoptosis-Related Protein Analysis

Each lysed sample from midbrain and striatum tissues from A53T and WT mice was used to investigate the apoptosis-related proteomic profile by a Proteome Profiler Mouse Apoptosis Array (R&D System, Minneapolis, MN, USA; ARY031), a membrane-based sandwich immunoassay. Apoptosis-related proteins were visualized by chemiluminescence (GE Healthcare, Milan, Italy), and levels were quantified with ImageJ software (NIH, Bethesda, MD, USA).

### 2.5. Intracellular ROS Release Measurement by Cytofluorimetry

The generation of ROS was estimated in primary A53T and WT midbrain and striatal astrocytes using the fluorescent probe 2′,7′-dichlorofluorescein-diacetate (H_2_DCF-DA). After reaching the exponential phase of growth, cells were collected, washed twice, and then incubated in PBS containing H_2_DCF-DA (10 μM) at 37 °C for 30 min [35]. In the presence of intracellular ROS, H_2_DC, derived from H_2_DCF-DA cleavage by intracellular esterases, was rapidly oxidized to the highly fluorescent 2′,7′-dichlorofluorescein (DCF). Flow cytometry evaluation was performed using the ATTUNE NxT acoustic focusing cytometer (Life Technologies). Data analysis was performed using the FlowJo Software (FlowJo, LLC). The intracellular ROS levels were expressed as mean fluorescence intensity.

### 2.6. Electrophysiology

K^+^ currents were recorded in A53T and WT primary astrocytes from the midbrain and striatal by the patch-clamp technique in a whole-cell configuration using a commercially available amplifier Axopatch 200B and a Digidata 1322A interface (Molecular Devices, San Jose, CA, USA), as previously described [29,31,32,36,37]. Spontaneous action potential (AP) activity was measured in A53T and WT primary astrocytes from the midbrain and striatal using the protocol previously described [32].

### 2.7. Ca^2+^_i_ Measurement

Intracellular calcium (Ca^2+^_i_) was measured by means of single-cell Fluo-4 acetoxymethyl-ester (AM) video imaging [38,39]. Fluo-4 AM was prepared as 1 mM stock solution in labeling grade DMSO and used at the final concentration of 10 μM. Astrocytes from different brain areas of A53T mice, placed on glass coverslips, were loaded with this probe for 30 min at 37 °C in normal Krebs solution containing 5.5 mM KCl, 160 mM NaCl, 1.2 mM MgCl_2_, 1.5 mM CaCl_2_, 10 mM glucose, and 10 mM HEPES-NaOH (pH 7.4). The live-imaging system was composed of an inverted Zeiss Axiovert 200 microscope (Carl Zeiss, Goettingen, Germany), a MicroMax 512BFT cooled CCD camera (Princeton Instruments, Trenton, NJ, USA), a LAMBDA10-2 filter wheeler (Sutter Instruments, Novato, CA, USA), and Meta-Morph/MetaFluor Imaging System software version 7.8 (Universal Imaging, West Chester, PA, USA). Astrocytes were illuminated at 494 nm, while the emission λ was 506 nm. Ca^2+^ release from ER stores was stimulated with the rapid addition of the SERCA pump inhibitor thapsigargin (TG, 1 μM) in a Krebs-Ringer saline solution.

### 2.8. Data and Statistical Analysis

Data are reported as mean ± S.E.M. of the values obtained from individual experiments. One-way analysis of variance (ANOVA) and Bonferroni’s test were used for data analysis to perform statistical comparisons between groups using GraphPad Prism 5.03 (GraphPad Software, La Jolla, CA, USA) for statistical analyses. A *p* value less than 0.05 was considered significant.

## 3. Results

### 3.1. Time Dependent Correlation among K_V_3.4 Channel and p27^kip1^, XIAP, and Smac/DIABLO in A53T Midbrain and Striatum

Starting from the previous reports demonstrating that the K_V_3.4 channel is precociously upregulated in AD astrocytes [30,32], we evaluated the possible mechanistic role of K_V_3.4 channels in PD progression. To this aim, we performed Western blotting experiments on the lysed midbrain and striatum collected from 4-, 12-, and 16-month-old A53T mice, a well-known familial PD model overproducing α-syn [33], and age-matched WT mice. As expected, Western blotting analysis revealed that TH was significantly downregulated in the lysed midbrain collected from 4- and 12-month-old A53T mice compared with respective age-matched WT (Figure S1). On the other hand, TH protein expression started to decrease at 12 months in the A53T striatum compared with age-matched WT, whereas it did not change at 4 months (Figure S1).

**Figure 1:**
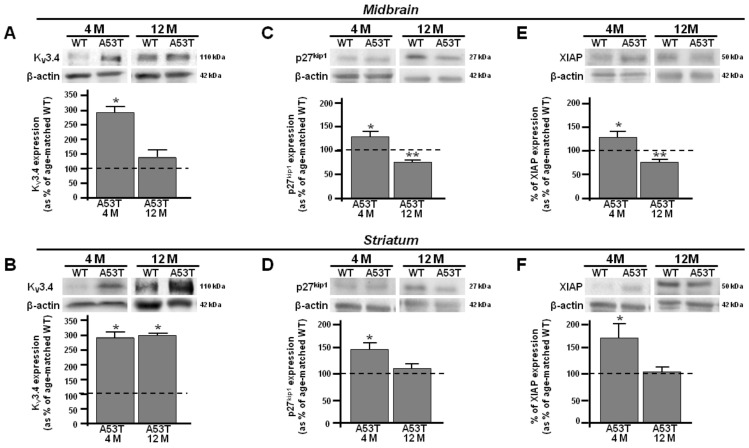
K_V_3.4, p27^kip1^, and XIAP protein expression in midbrain and striatum obtained from 4- and 12-month-old A53T and WT mice. (**A**) Representative Western blotting (upper) and densitometric quantification (lower) of K_V_3.4 protein expression in A53T and WT midbrain at 4 and 12 months. (**B**) Representative Western blotting (upper) and densitometric quantification (lower) of K_V_3.4 protein expression in A53T and WT striatum at 4 and 12 months. (**C**) Representative Western blotting (upper) and densitometric quantification (lower) of p27^kip1^ protein expression in A53T and WT midbrain at 4 and 12 months. (**D**) Representative Western blotting (upper) and densitometric quantification (lower) of p27^kip1^ protein expression in A53T and WT striatum at 4 and 12 months. (**E**) Representative Western blotting (upper) and densitometric quantification (lower) of XIAP protein expression in A53T and WT midbrain at 4 and 12 months. (**F**) Representative Western blotting (upper) and densitometric quantification (lower) of XIAP protein expression in A53T and WT striatum at 4 and 12 months. Number of animals at 4 months, WT #7 and A53T #5, and at 12 months, WT #5 and A53T #5. Each bar represents the mean% ± S.E.M. of different experimental values obtained in 3 independent experimental sessions. * *p* < 0.05 compared to age-matched WT midbrain and striatum; ** *p* < 0.05 compared to age-matched A53T midbrain and striatum.

**Figure 2:**
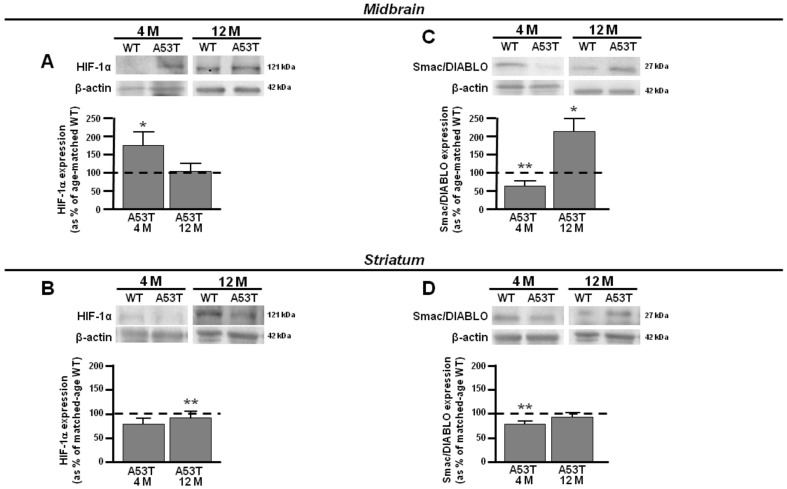
HIF-1α and Smac/DIABLO protein expression in midbrain and striatum obtained from 4- and 12-month-old A53T and WT mice. (**A**) Representative Western blotting (upper) and densitometric quantification (lower) of HIF-1α protein expression in A53T and WT midbrain at 4 and 12 months. (**B**) Representative Western blotting (upper) and densitometric quantification (lower) of HIF-1α protein expression in A53T and WT striatum at 4 and 12 months. (**C**) Representative Western blotting (upper) and densitometric quantification (lower) of Smac/DIABLO protein expression in A53T and WT midbrain at 4 and 12 months. (**D**) Representative Western blotting (upper) and densitometric quantification (lower) of Smac/DIABLO protein expression in A53T and WT striatum at 4 and 12 months. Number of animals at 4 months, WT #7 and A53T #5, and at 12 months, WT #5 and A53T #5. Each bar represents the mean% ± S.E.M. of different experimental values obtained in 3 independent experimental sessions. * *p* < 0.05 compared to age-matched WT midbrain and striatum; ** *p* < 0.05 compared to age-matched A53T midbrain and striatum.

Densitometric analysis revealed an early upregulation of the K_V_3.4 channel in the 4-month-old A53T midbrain and striatum (Figure 1A,B). The band of K_V_3.4 channels at 110 kDa was overexpressed in the A53T midbrain and striatum at 4 months compared with age-matched controls (Figure 1A,B). In addition, the expression of K_V_3.4 channels was downregulated at 12 months in the A53T midbrain (Figure 1A), whereas it was still overexpressed at 12 months in the A53T striatum compared with the respective age-matched WT (Figure 1B). Of note, the early overexpression of K_V_3.4 channels, observed in the A53T midbrain and striatum at 4 months, matched with the upregulation of the cyclin-dependent kinase inhibitor 1B (p27^Kip1^), a negative regulator of CDK/cyclins protecting cells from apoptosis, and X-linked-inhibitor of apoptosis protein (XIAP), a direct inhibitor of cell-death proteases [40,41], compared to the age-matched congenic WT (Figure 1C–F). Moreover, Western blot analysis revealed a selective upregulation of the band at ~121 kDa, corresponding to the hypoxia inducible factor-1α (HIF-1α), a key regulator of the cellular response to hypoxia [42], in the A53T midbrain, which did not change in the A53T striatum at 4 months (Figure 2A,B). In addition, densitometric analysis revealed a downregulation of the band at ~27 kDa, corresponding to Smac/DIABLO, an apoptosis promoter, by neutralizing the inhibitor of apoptosis proteins (IAPs) [43] in the A53T midbrain and striatum compared with the respective age-matched WT (Figure 2C,D). At a subsequent time of 12 months, the A53T midbrain revealed a downregulation of the bands at ~27 and ~50 kDa, corresponding to p27^Kip1^ and XIAP, respectively, and an upregulation of Smac/DIABLO compared to age-matched congenic WT, suggesting a switch from the anti- to pro-apoptotic phenotype (Figure 1C,E and Figure 2C). In addition, the expression of HIF-1α did not change in the A53T midbrain at 12 months (Figure 2A). On the other hand, at 12 months, the A53T striatum retained an antiapoptotic pathway (Figure 1D,F). Densitometric analysis showed that the expression of Smac/DIABLO, XIAP, and p27^Kip1^ did not change in the A53T striatum compared with age-matched WT at 12 months (Figure 1D,F and Figure 2D). Densitometric analysis showed that the expression of HIF-1α was not modified in the A53T striatum compared with age-matched WT at 12 months (Figure 2B).

### 3.2. Different Time-Dependent Proteome Profiler Displayed by A53T Midbrain and Striatum

**Figure 3:**
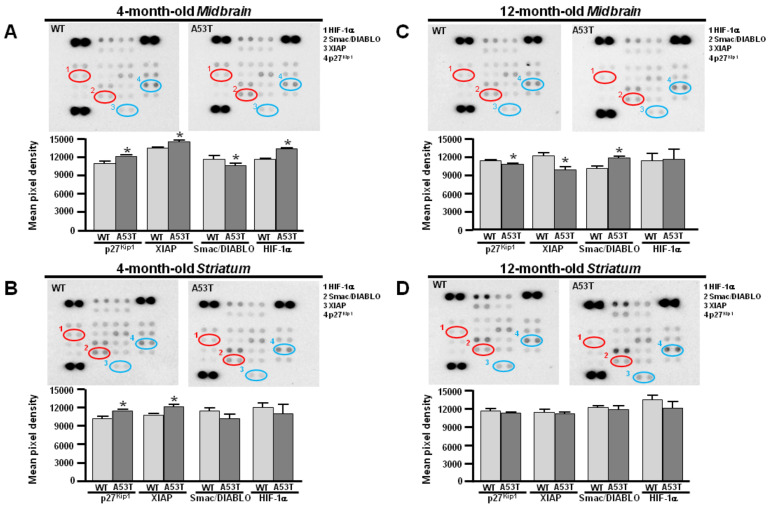
Proteomic immunoassay of p27^kip1^, XIAP, HIF-1α, and Smac/DIABLO protein expression in the midbrain and striatum obtained from 4- and 12-month-old A53T and WT mice. (**A**) Representative blots of proteomic immunoassay (upper) and densitometric quantification (lower) of (4) p27^kip1^, (3) XIAP (blue circles), (1) HIF-1α, and (2) Smac/DIABLO (red circles) protein expression in A53T and WT midbrain at 4 months. (**B**) Representative blots of proteomic immunoassay (upper) and densitometric quantification (lower) of p27^kip1^, XIAP, HIF-1α, and Smac/DIABLO protein expression in A53T and WT striatum at 4 months. (**C**) Representative blots of proteomic immunoassay (upper) and densitometric quantification (lower) of p27^kip1^, XIAP, HIF-1α, and Smac/DIABLO protein expression in A53T and WT midbrain at 12 months. (**D**) Representative blots of proteomic immunoassay (upper) and densitometric quantification (lower) of p27^kip1^, XIAP, HIF-1α, and Smac/DIABLO protein expression in A53T and WT striatum at 12 months. Number of animals at 4 months, WT #7 and A53T #5, and at 12 months, WT #5 and A53T #5. Each bar represents the mean% ± S.E.M. of different experimental values obtained in 3 independent experimental sessions. Anti-apoptotic proteins in blue circle and pro-apoptotic proteins in red circle * *p* < 0.05 compared to age-matched WT midbrain and striatum.

Consistently, the proteome profiler revealed an anti-apoptotic phenotype in both A53T midbrain and striatum at 4 months (Figure 3A,B). More specifically, densitometric analysis showed an upregulation of XIAP and p27^Kip1^ expression in the 4-month-old A53T midbrain and striatum compared with their age-matched congenic WT (Figure 3A,B). Consistently with previous results, HIF-1α was selectively overexpressed in the A53T midbrain, whereas it did not change in the A53T striatum at 4 months (Figure 3A,B). Of note, the expression of Smac/DIABLO was downregulated in the 4-month-old A53T midbrain and striatum compared to the respective age-matched WT (Figure 3A,B). At 12 months, the proteome profiler revealed that the A53T midbrain showed pro-apoptotic features (Figure 2C). Densitometric analysis revealed an overexpression of Smac/DIABLO and a downregulation of XIAP and p27^Kip1^, whereas HIF-1α did not change in the A53T midbrain at 12 months compared to age-matched WT (Figure 3C). Differently, A53T striatum retained an anti-apoptotic phenotype at 12 months (Figure 3D). Consistently, densitometric analysis revealed that the expression of XIAP was higher, whereas Smac/DIABLO started to increase compared with the age-matched WT (Figure 3D). In addition, the expression of p27^Kip1^ did not change in the A53T striatum at 12 months (Figure 3D).

**Figure 4:**
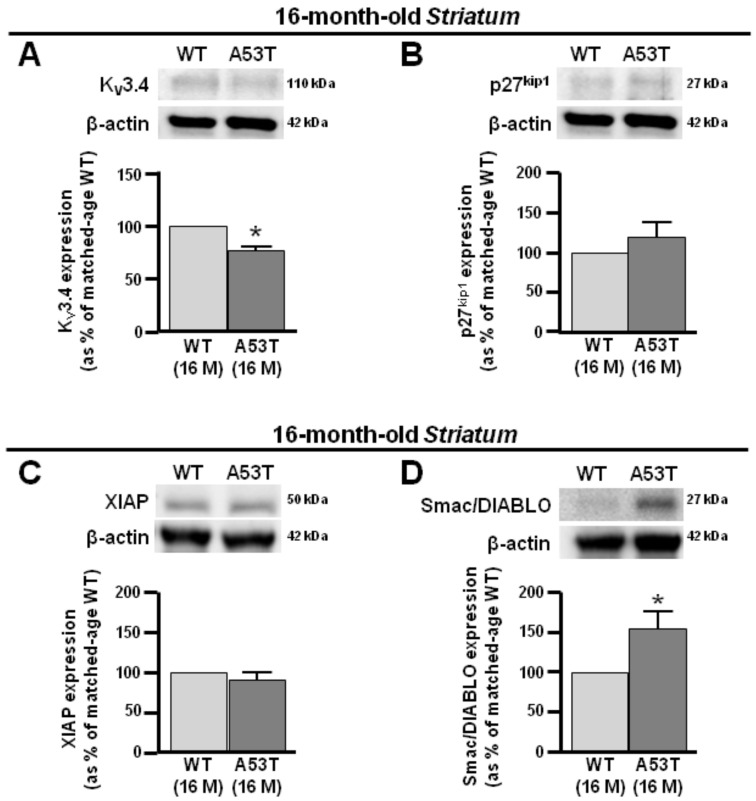
K_V_3.4, p27^kip1^, XIAP, and Smac/DIABLO protein expression in striatum obtained from 16-month-old A53T and WT mice. (**A**) Representative Western blotting (upper) and densitometry quantification (lower) of K_V_3.4 protein expression in A53T and WT striatum at 16 months. (**B**) Representative Western blotting (upper) and densitometry quantification (lower) of p27^kip1^ protein expression in A53T and WT striatum at 16 months (**C**) Representative Western blotting (upper) and densitometry quantification (lower) of XIAP protein expression in A53T and WT striatum at 16 months. (**D**) Representative Western blotting (upper) and densitometry quantification (lower) of Smac/DIABLO protein expression in A53T and WT striatum at 16 months. Number of animals at 16 months, WT #5 and A53T #5. Each bar represents the mean% ± S.E.M. of different experimental values obtained in 3 independent experimental sessions. * *p* < 0.05 compared to age-matched WT midbrain and striatum.

Remarkably, the A53T striatum started to assume a pro-apoptotic phenotype, 4 months later than the A53T midbrain at 16 months (Figure 4). At this time, densiometric analysis revealed that the p27^Kip1^ and XIAP expression decreased, whereas Smac/DIABLO was overexpressed (Figure 4B,C). Interestingly, K_V_3.4 protein expression was downregulated in the A53T striatum at 16 months (Figure 4A). These results seemed to suggest the involvement of K_V_3.4 channel downregulation in the switch from the anti- to pro-apoptotic pathway. Remarkably, the WT midbrain and striatum at 12 and 16 months, respectively, showed an overexpression of K_V_3.4 channel subunits compared to the respective WT at 4 months.

### 3.3. Different Modulation of ROS-Dependent K_V_3.4 Channel in Primary A53T Midbrain and Striatum Astrocytes

**Figure 5:**
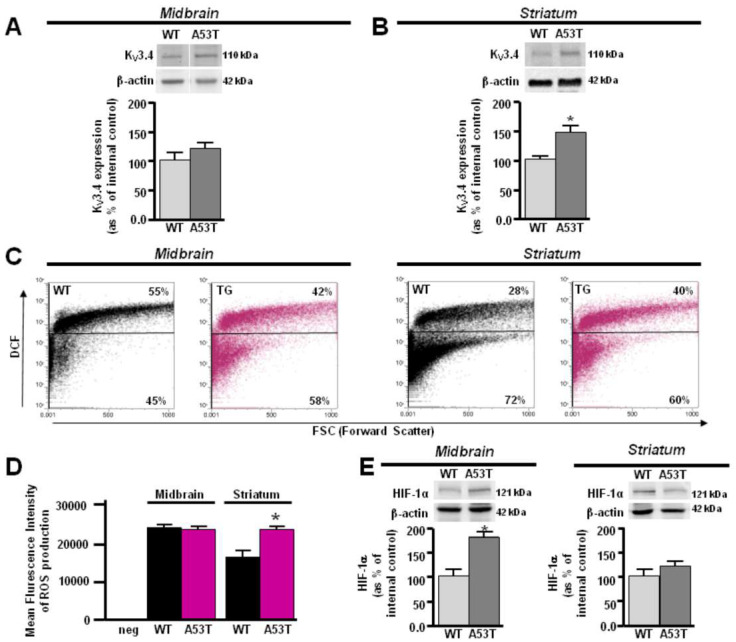
Different modulation of ROS-dependent K_V_3.4 channels in primary A53T midbrain and striatum astrocytes. (**A**) Representative Western blotting and densitometry quantification of K_V_3.4 protein expression in primary A53T and WT astrocytes from midbrain. (**B**) Representative Western blotting and densitometry quantification of K_V_3.4 protein expression in primary A53T and WT astrocytes from striatum. Each bar represents the mean ± S.E.M. of the percentage of different experimental values obtained in three independent experimental sessions. (**C**) Representative dot plots of DCF density in primary A53T (magenta) and WT astrocytes (black) from striatum (left) and midbrain (right). (**D**) Mean Fluorescence Intensity of ROS production in C panel. Each bar represents the mean ± S.E.M. of the DCF fluorescence of different experimental values obtained in three independent experimental sessions. (**E**) Representative Western blotting and densitometry quantification of HIF-1α protein expression in primary A53T and WT astrocytes from midbrain and striatum. Each bar represents the mean% ± S.E.M. of different experimental values obtained in three independent experimental sessions. * *p* < 0.05 compared to midbrain and striatum WT astrocytes.

**Figure 6:**
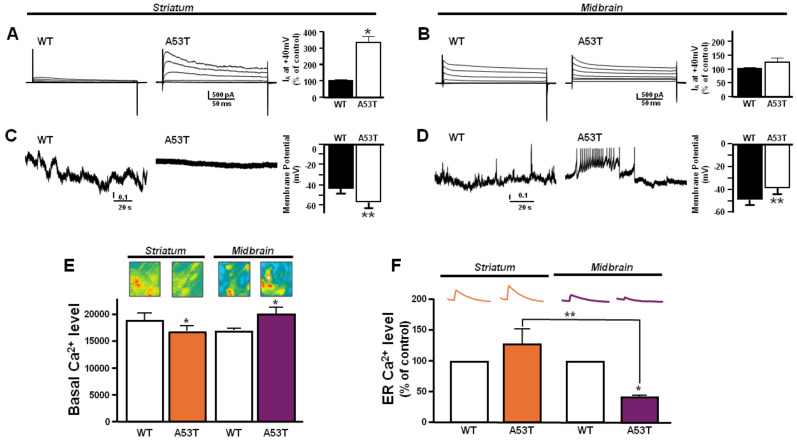
Effect of the activity of K_V_3.4 channels on resting membrane potentials and [Ca^2+^]_i_ transients in primary A53T and WT astrocytes from midbrain and striatum. (**A**) Representative traces (left) and quantification of K_V_3.4-mediated fast inactivating K^+^ currents (I_A_; right) recorded from primary A53T and WT astrocytes from striatum. (**B**) Representative traces (left) and quantification of K_V_3.4-mediated I_A_ currents (right) recorded from primary A53T and WT astrocytes from midbrain. The peak values of I_A_ currents, measured at the beginning of the +40 mV depolarizing pulse, are expressed as mean% ± SEM of 3 independent experiments performed on 3 different preparations (for both (**A**,**B**): n = 12 cells in each cell culture and for each group). (**C**) Representative traces recorded in the gap-free mode (left) and quantification of membrane resting potential (right) in A53T and WT astrocytes from striatum. (**D**) Representative traces recorded in the gap-free mode (left) and its quantification (right) in A53T and WT astrocytes from midbrain. Vertical scale bar below the trace represents 1 pA. The values are expressed as mV and represent the mean ± SEM of 3 independent experiments performed on 3 different preparations (for both (**C**,**D**): n = 12 cells for each group). (**E**) Quantification of Ca^2+^_i_ in A53T and WT astrocytes from striatum and midbrain loaded with Fluo-4AM; * *p* < 0.05 vs. its respective WT. (**F**) Quantification of ER Ca^2+^ in A53T and WT astrocytes from striatum and midbrain loaded with Fluo-4AM; * *p* < 0.05 vs. WT astrocytes from midbrain. ** *p*< 0.05 A53T astrocytes from midbrain vs. A53T astrocytes from striatum

Consistently with previous in vivo results, primary striatum astrocytes from A53T mice showed a pronounced immunoreactivity of the band at 110 kDa corresponding to the K_V_3.4 channel (Figure 5B). On the other hand, primary astrocytes from the A53T midbrain displayed only a slight increase in K_V_3.4 protein expression compared with control astrocytes (Figure 5A). The flow cytometry experiments showed a different intracellular ROS production in the primary A53T midbrain and striatum astrocytes (Figure 5C,D). Cytofluorimetric analysis revealed a higher percentage of ROS-producing astrocytes in the A53T striatum than in its control (Figure 5C). Consistently, the mean fluorescence intensity of ROS production was increased in A53T astrocytes compared to WT astrocytes in the striatum (Figure 5D). On the other hand, no significant changes were observed between A53T and WT midbrain astrocytes (Figure 5C,D). However, the percentage of ROS-producing astrocytes and the mean fluorescence intensity of ROS production in A53T and WT midbrain astrocytes were comparable to A53T striatum astrocytes (Figure 5C,D). Of note, in line with in vivo results, Western blot analysis revealed that HIF-1α was overexpressed in A53T midbrain astrocytes compared with WT, whereas it did not change in A53T striatum astrocytes (Figure 5E). In addition, patch-clamp recordings revealed that primary A53T striatum astrocytes showed hyperpolarized resting membrane potentials and lower firing frequency associated with K_V_3.4 hyperactivity compared with primary littermate astrocytes (Figure 6A). By contrast, primary A53T midbrain astrocytes displayed a depolarized resting membrane potential and higher firing frequency accompanied by a slight increase of K_V_3.4 currents compared with primary littermate astrocytes (Figure 6B). Intracellular calcium level (Ca^2+^_i_) measurements were in line with the resting membrane potential data obtained in A53T astrocytes. In fact, primary A53T midbrain astrocytes displayed higher Ca^2+^_i_ than the respective primary littermate astrocytes (Figure 6E) as a result of the modest shift of their membrane potential toward more depolarizing values. However, primary A53T striatum astrocytes showed a lower Ca^2+^_i_ compared with their primary littermate astrocytes (Figure 6E), displaying a hyperpolarizing shift of its membrane potential. Moreover, Ca^2+^_i_ released from ER by the ER releasing agent TG was significantly lower in primary A53T midbrain astrocytes compared with the respective littermate controls and A53T striatum astrocytes, thereby suggesting the occurrence in the midbrain of a significant ER Ca^2+^ depletion (Figure 6F).

## 4. Discussion

The present study provides evidence that the K_V_3.4 channel, a ROS-sensitive channel carrying a fast-inactivating current, takes part in the different time-dependent vulnerabilities of the midbrain and striatum during PD progression. The present findings were obtained in a genetic model carrying one of the myriad of human mutations expressed by PD patients. Therefore, it is difficult to trace back to human disease. However, mice carrying the A53T mutation provide a widely used preclinical model to identify pathomechanisms of the disease. Particularly, we observed an early overexpression of K_V_3.4 channels in the A53T midbrain and striatum at 4 months that matched with an antiapoptotic phenotype characterized by the overexpression of p27^kip1^ and XIAP, two negative regulators of apoptosis [40,41]. Of note, it has been demonstrated in PD that p27^kip1^, acting as a negative regulatory mechanism of α-syn expression, exerts a neuroprotective action, preventing accumulation and aggregation of α-syn [44]. In addition, the upregulation of XIAP, a member of the IAP family, exerts a neuroprotective action [45,46]. In the present study, we showed an early upregulation of the K_V_3.4 channel that matched with a downregulation of Smac/DIABLO, a positive regulator of apoptosis in several neurodegenerative diseases [43]. Consistently, epigallocatechin-3-gallate, the main ingredient of green tea polyphenols, downregulates Smac/DIABLO, restoring the mitochondrial membrane potential and exerting a neuroprotective action in PD [47]. Furthermore, delayed release of Smac/DIABLO by Hsp27 overexpression reduces caspase activity and apoptosis in a model of PD [48].

These results suggest that the overexpression of the K_V_3.4 channel could play an early neuroprotective role in both midbrain and striatum PD. Interestingly, at 12 months, the expression of the K_V_3.4 channel was differently modulated in the A53T midbrain and striatum. Particularly, the K_V_3.4 channel was downregulated in the A53T midbrain, whereas it remained overexpressed in A53T striatum. Notably, at 12 months, the A53T midbrain displayed a suffering phenotype, switching from anti- to pro-apoptotic features, thus showing a downregulation of p27^kip1^ and XIAP and an upregulation of Smac/DIABLO. Conversely, in the A53T striatum, at the same time, the K_V_3.4 channel expression remained upregulated, retaining an anti-apoptotic phenotype. Indeed, the expression of p27^kip1^ and XIAP was still overexpressed, as highlighted by proteomic and Western blot analyses. In line with the hypothesis of the major vulnerability of midbrain PD, the A53T midbrain displayed a selective overexpression of HIF-1α, whereas its expression did not change in the A53T striatum at the same time. HIF-1α, the master transcriptional regulator of cells’ response to hypoxia, is upregulated in the injured brain, highlighting its detrimental role [42]. Therefore, the upregulation of HIF-1α could be considered as a prelude to the switch from an anti- to a pro-apoptotic phenotype. These observations seem to strongly suggest that the expression of K_V_3.4 channels could play a role in the different vulnerabilities between midbrain and striatum and that overexpression might play a neuroprotective role in PD progression in animal models.

It should be noted that at 16 months, A53T striatum switched from the anti- to pro-apoptotic phenotype, displaying an upregulation of Smac/DIABLO and a downregulation of p27^kip1^ and XIAP levels.

Therefore, the onset of pro-apoptotic pathways appeared 4 months later in the striatum than midbrain, in correspondence with the downregulation of K_V_3.4 expression.

Evidence that the midbrain and striatum are differently involved in PD pathogenesis is supported by data showing that cellular damage starts in the midbrain of PD patients as a precocious event [3,4]. Then, this mechanism can stimulate the activation of glial cells in the striatum [2]. Molecularly, it has been observed that a progressive mitochondrial dysfunction, occurring in the early stage of PD in the midbrain, could generate dopaminergic neuronal damage responsible for the late PD progression [2,49,50]. Moreover, in the present study, a new role is attributed to the K_V_3.4 channel in PD progression. Similarly, the upregulation of the K_V_3.4 channel has been proposed as one of the early events occurring before the appearance of amyloid plaques and neurofibrillary tangles in the human AD brain [27]. However, K_V_3.4 channels are also expressed in the processes of astrocytes [51], where the functional implications have not been fully elucidated. For instance, K_V_ channels’ blockade in cortical astrocytes reduces cell repolarization [21]. In this regard, the electrophysiological results of the present study suggest that the upregulation of the K_V_3.4 channel in primary A53T striatum astrocytes was associated with a hyperpolarized resting membrane potential and a consequent lower Ca^2+^_i_ level than the primary littermate astrocytes. These could be considered two important factors in regulating resting astrocyte function [21]. On the other hand, primary A53T midbrain astrocytes, showing a downregulation of this channel, displayed a depolarized resting membrane potential and a dysfunctional Ca^2+^ homeostasis, two predictive factors of vulnerability [32]. Furthermore, it has been established that K_V_3.4 channel activity could be inhibited by HIF-1α, which is able to mirror the action of the BDS-I, a well-documented K_V_3.4-selective blocker [26]. In consideration of the inhibitory role played by HIF on Kv3.4 expression and activity [26], preliminary data showed that a detrimental pathway (i.e., upregulation of Smac/DIABLO/downregulation of p27kip1, XIAP, and BCl2) was activated in midbrain astrocytes of A53T mice expressing high levels of the transcription factor.In fact, under hypoxic conditions, the overexpression of HIF-1α is concomitant to a downregulation of K_V_3.4 activity, despite its overexpression [52,53,54]. Of interest, biochemical analysis showed a selective overexpression of HIF-1α in A53T midbrain astrocytes but not in A53T striatum astrocytes, suggesting the greater vulnerability of the midbrain compared to the striatum.

From the molecular point of view, there is similarity to AD mechanisms, since the upregulation of the K_V_3.4 channel appears to be mediated by the activation of nuclear factor κB-dependent (NFκB) pathway [28,29,30]. In this respect, the NF-κB transcription factor family in mammals consists of five proteins, of which p65 (RelA) and c-Rel have a specific role in PD pathogenesis. In fact, inhibition of RelA seems to exert neuroprotection against 1-methyl-4-phenyl-1,2,3,6-tetrahydropyridine (MPTP) and 1-methyl-4-phenylpyridinium (MPP+) toxicity, suggesting that this factor decreases neuronal resilience. Conversely, the c-Rel subunit can exert neuroprotective actions [55,56]. In support of this view, a significant reduction in c-Rel expression in whole blood samples from PD patients has been reported [57], thus supporting the idea of the loss of the protective role of NF-kB during PD-related neurodegeneration. As concerns PD models, at 18 months of age, c-Rel KO mice also displayed a striatal increase in the pro-apoptotic form of RelA [58], supporting the induction of neuronal damage in this brain area. Furthermore, RelA has been detected in the nucleus of subpopulations of neurons and glial cells of the *substantia nigra* of PD patients [59,60]. Furthermore, the nuclear content of RelA is abnormally increased in nigral dopamine neurons and glial cells of PD patients [61]. In accordance with these data, a decreased expression of the NF-κB inhibitor A20 has been found in whole blood from PD patients [62].

Therefore, the different modulation of NF-kB and HIF-1α in the midbrain and striatum, modulating K_V_3.4 channel activity, might be responsible for the different vulnerability of the two areas.

In this respect, several excellent studies have identified differences in the expression level of several proteins, such as calcium binding proteins, metal transporters, and antioxidant enzymes in different midbrain dopaminergic regions within the mammalian brain, which may contribute to the relative vulnerability of the *substantia nigra pars* compacta in PD [63]. Recently, K^+^ channels are emerging among the factors within the substantia *nigra pars compacta* on postmortem brain areas, which may influence neuronal vulnerability, but this has not yet been comprehensively examined. Interesting, an overexpression of GIRK2, a G-protein-coupled inward rectifying current potassium channel type-2, has been observed in the postmortem midbrain from PD patients compared with the age-matched control [64]. In addition, immunoanalysis has revealed a co-expression of GIRK2/tyrosine hydroxylase TH [64]. Of note, a transcriptional upregulation of the KATP channel in surviving *substantia nigra* DA neurons from PD patients has been assessed, suggesting that KATP might contribute to the pathophysiology of human substantia nigra DA [65]. Recently, Sarkar et al. [66] observed an overexpression of the K_V_1.3 channel in the *substantia nigra* from animal models of PD, as well as from postmortem human PD brains [66]. In addition, an involvement of the large conductance calcium-activated potassium (BK) channels in the pathogenesis of PD has been indirectly demonstrated. In particular, LINGO1, a transmembrane protein regulating BK channels, is upregulated in the cerebellum of PD patients [67].

Collectively, despite the limitative lack of similar data obtained in human samples, these preclinical findings suggest that the early K_V_3.4 overexpression could take part not only in PD pathogenesis but also in the different vulnerability of the midbrain and striatum, highlighting K_V_3.4 as a possible new therapeutic target in PD.

## Data Availability

The data presented in this study are available on request from the corresponding author due to privacy.

## Supplementary Materials

The following supporting information can be downloaded at: https://www.mdpi.com/article/10.3390/antiox13080999/s1.
