# Supplementary material for: Involvement of KV3.4 Channel in Parkinson’s Disease: A Key Player in the Control of Midbrain and Striatum Differential Vulnerability during Disease Progression?
Source: Antioxidants (Basel). 2024 Aug 18;13(8):999. doi: 10.3390/antiox13080999 (PMC11351402; doi:10.3390/antiox13080999)
Supplement: Supplementary file 1 [file antioxidants-13-00999-s001.zip › antioxidants-3150572-supplementary.pdf]

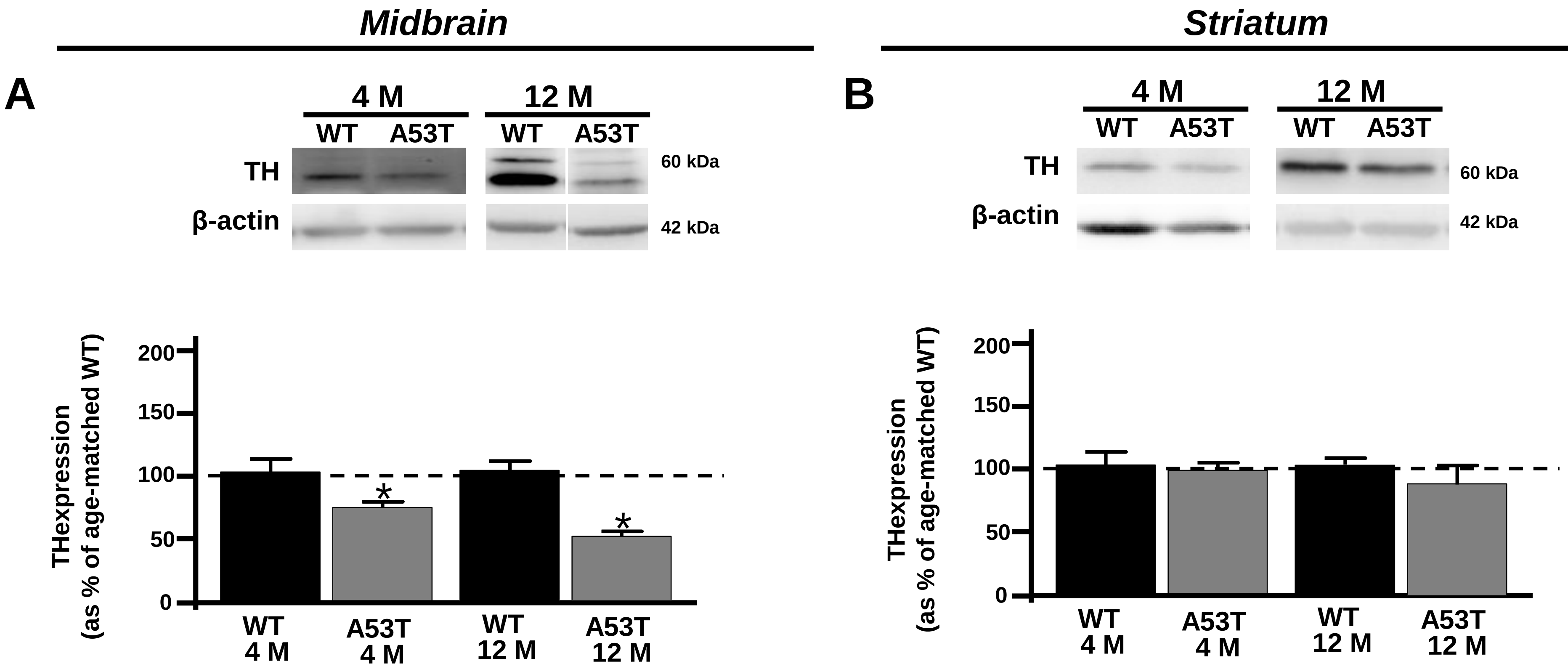

Figure S1. TH protein expression in midbrain and striatum obtained from 4- and 12-month-old A53T and WT mice. (A) Representative Western blotting (upper) and densitometric quantification (down) of TH protein expression in A53T and WT midbrain at 4 and 12 months. (B) Representative Western blotting (upper) and densitometric quantification (down) of TH protein expression in A53T and WT striatum at 4 and 12 months. Number of animals at 4 months WT #7 and A53T #5 and at 12 months WT #5 and A53T #5. Each bar represents the mean ± S.E.M. of the percentage of different experimental values obtained in three independent experimental sessions. *$p < 0.05$ compared to age-matched WT midbrain and striatum.

Figure S1
